# Vulvar cancer staging: guidelines of the European Society of Urogenital Radiology (ESUR)

**DOI:** 10.1186/s13244-021-01075-6

**Published:** 2021-09-22

**Authors:** Olivera Nikolić, Filipa Alves e Sousa, Teresa Margarida Cunha, Marijana Basta Nikolić, M. Milagros Otero-García, Benedetta Gui, Stephanie Nougaret, Henrik Leonhardt, On behalf of the ESUR Female Pelvic Imaging Working Group, Stephanie Nougaret, Laure Fournier, Charis Bourgioti, Athina C. Tsili, Milagros Otero-Garcia, Lucia Manganaro, Teresa Margarida Cunha, Aki Kido, Celine Alt, Rita Lucas, Henrik Leonhardt, Benedetta Gui, Rosemarie Forstner, Cristina Maciel, Evis Sala, Nishat Bharwani, Laura Buñesch, Yulia Lakhman, Carolina Lopez, Olivera Nikolic, Marijana Basta Nikolić

**Affiliations:** 1 Center of Radiology, Clinical Center of Vojvodina, Faculty of Medicine, University of Novi Sad, Hajduk Veljkova 1-9, 21000 Novi Sad, Serbia; 2 Department of Radiology, Centro Hospitalar Universitário de Lisboa Central, Alameda Santo António Dos Capuchos, 1169-050 Lisboa, Portugal; 3 Department of Radiology, Instituto Português de Oncologia de Lisboa Francisco Gentil, R. Prof. Lima Basto, 1099-023 Lisbon, Portugal; 4 Hospital Universitario de Vigo (CHUVI), Estrada Clara Campoamor 341, 36312 Vigo, Spain; 5 Fondazione Policlinico Universitario A. Gemelli IRCCS, UOC Radiologia Generale Ed Interventistica Generale, Area Diagnostica Per Immagini, Dipartimento Diagnostica Per Immagini, Radioterapia Oncologica ed Ematologia, Rome, Italy; 6 Department of Radiology, Montpellier Cancer Institute, 15 INSERM, Montpellier Cancer Research Institute, U1194, University of Montpellier, 208 Avenue des Apothicaires, 34295 Montpellier, France; 7 Department of Radiology, Institute of Clinical Sciences, Sahlgrenska University Hospital, Bruna straket 11B, 413 45 Gothenburg, Sweden

**Keywords:** Vulvar cancer, Staging, Magnetic resonance imaging, Guidelines, Protocol

## Abstract

**Objective:**

The aim of the Female Pelvic Imaging Working Group of the European Society of Urogenital Radiology (ESUR) was to develop imaging staging guidelines for vulvar cancer and to propose standardised MRI protocols and reporting.

**Methods:**

The guidelines recommended from the ESUR in this article resulted from a questionnaire analysis regarding imaging staging of vulvar cancer that was answered by all members of the Female Pelvic Imaging Working Group. Only the answers with an agreement equal to or more than 80% were considered. Additionally, the literature was reviewed to complement and further support our conclusions.

**Results:**

The critical review of the literature and consensus obtained among experts allows for recommendations regarding imaging staging guidelines, patient preparation, MRI protocol, and a structured MRI report.

**Conclusions:**

Standardising image acquisition techniques and MRI interpretation reduces ambiguity and ultimately improves the contribution of radiology to the staging and management of patients with vulvar cancer. Moreover, structured reporting assists with the communication of clinically relevant information to the referring physician.

## Key points

- MRI is the modality of choice for local staging of vulvar cancer.
- T2WI, DWI-MRI, and DCE-MR are recommended.
- The most widely accepted criterion for inguinofemoral lymphadenopathy is short-axis > 1 cm.
- The most specific criterion for inguinofemoral lymphadenopathy is the presence of necrosis.

## Introduction

Vulvar cancer is a rare gynaecologic malignancy, representing only 2–5% of cases, primarily affecting postmenopausal women [1]. Initial diagnosis is made by gynaecological examination and punch/incision biopsy. Squamous cell carcinomas (SCC) account for the vast majority of vulvar cancers (> 85%) [2]. The International Federation of Gynaecology and Obstetrics (FIGO) [3] and the TNM classification [4] systems are both used to stage vulvar cancer and are closely aligned. The final diagnosis is established by histological examination of the primary tumour and lymph node specimens [5].

In vulvar cancer, metastatic involvement of the inguinofemoral lymph nodes is the most important prognostic factor and influences the surgical approach and the need for chemoradiation therapy [1, 6].

Imaging modalities, such as ultrasound, computed tomography (CT), combined ^18^F fluorodeoxyglucose positron emission tomography and CT (FDG-PET/CT), and magnetic resonance imaging (MRI), are not an integrated part of staging according to FIGO, nevertheless they are well-recognised to provide valuable information concerning local tumour status, lymphadenopathy, and distant metastasis. Clinical or imaging suspicion of lymph node involvement should be further analysed by fine-needle aspiration (FNA) or core biopsy whenever this additional information impacts the primary treatment choice [5].

Due to its excellent contrast resolution, MRI is considered the imaging modality of choice for evaluating local growth of vulvar cancer and to exclude invasion of nearby situated organs. In spite of that MRI staging of vulvar cancer is not used routinely in all cancer centres, and it could be argued that MRI is only indicated for larger tumours [7]. Furthermore, despite its wide utilisation, a lack of standardised recommendations/guidelines for MR protocol and reporting is notable.

The aim of this manuscript is to present the ESUR recommendations for the initial staging of vulvar cancer, based on recent clinical and imaging developments. The value of an appropriate MR imaging protocol and standardised imaging reports is highlighted.

These guidelines apply to adults over the age of 18 who have SCC of the vulva and do not address patients with other vulvar cancer histologies.

## Material and methods

### Questionnaire and consensus meeting

A Questionnaire consisting of 54 questions was designed by the authors and then sent out to the Female Pelvic Imaging Working Group for approval. Indications and technical details, including minimal hardware characteristics, patient preparation, examination protocol and reporting were analysed. For some questions multiple answers were possible. Not all the questions were answered by all the participants. A total of 21 responses were obtained and analysed. Each item was classified as follows: (1) “RECOMMENDED” (at least 80% agreement in favour), (2) “NOT RECOMMENDED” (at least 80% agreement in opposition) or (3) “UNCERTAIN”, i.e. consensus was not reached (less than 80% agreement). The results were presented to and discussed with the ESUR Female Pelvic Imaging Working Group. The panel included 21 experts from 20 different institution in Europe: Portugal (*n* = 3), France (*n* = 2), Spain (*n* = 2), United Kingdom (*n* = 3), Germany (*n* = 1), Austria (*n* = 1), Sweden (*n* = 1), Italy (*n* = 2), Serbia (*n* = 2), and Greece (*n* = 2). Two panelists were from two institutions outside Europe: Japan (*n* = 1) and USA (*n* = 1). The panel’s recommendations (based on at least 80% consensus among experts) are given in Table 2.

### Literature search

We searched the PubMed/Medline database, using the following search terms: vulvar cancer; vulvar carcinoma; gynaecologic malignancies; ultrasound; computed tomography; magnetic resonance imaging; and positron-emission tomography/computed tomography (PET/CT). We selected relevant English-language papers on vulvar cancer, with a special focus on its imaging evaluation.

## Role of imaging in staging vulvar cancer

### Primary tumour staging

**Fig. 1:**
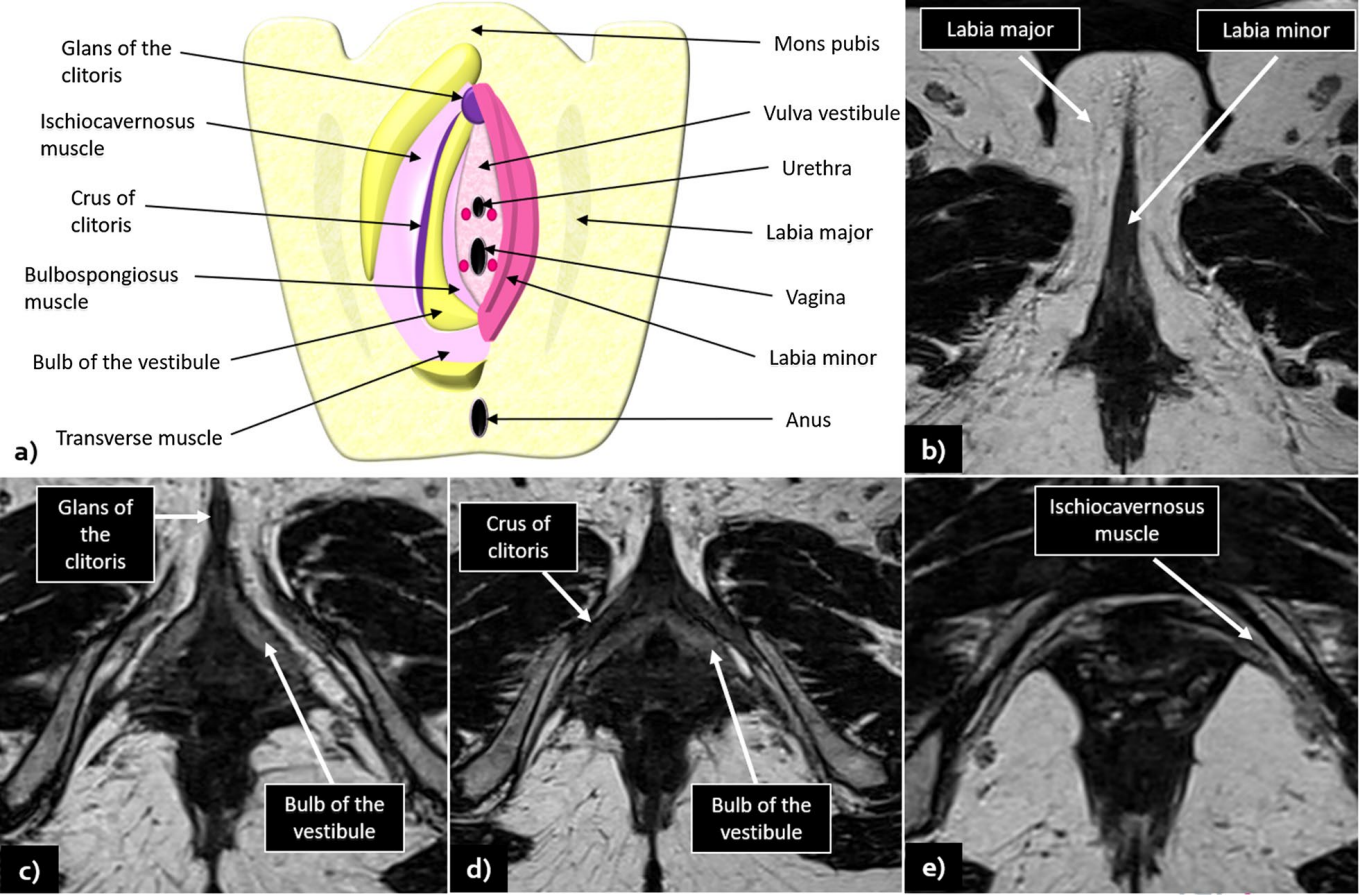
**a** Schematic illustration of vulvar anatomy: **b**–**e** show MRI normal findings and vulvar anatomy in axial T2WI sequences

Due to its excellent contrast resolution and the ability to depict perineal and vulvar anatomy to great detail (illustrated in Fig. 1), MRI is the imaging modality of choice for the local staging of vulvar cancer.

No relevant literature was found regarding MRI in the evaluation of primary tumours ≤ 2 cm, confined to the vulva and/or perineum, and with ≤ 1 mm of stromal invasion. As such, MRI is not recommended in these cases.

Pelvic MRI including the inguinal regions should be performed for local staging of SCC with stromal invasion > 1 mm, tumour size > 4 cm or tumours with suspicious involvement of the urethra, vagina, or anus according to clinical evaluation [8, 9]. A lack of evidence regarding the appropriateness of MRI staging of tumours sized between > 2 cm and ≤ 4 cm with stromal invasion ≤ 1 mm is noted and, in those cases, the decision to refer the patient to MRI should depend on the clinical suspicion of tumour invasion of the nearby situated organs.

In a study including 22 patients prior to surgery, MRI accurately staged primary tumour extent (T stage) in 70% of patients (Sohaib et al. [10]). In another study, tumour size was correctly assessed in 86% of cases, with both unenhanced and contrast-enhanced MRI, and the overall staging accuracy was 69.4% for unenhanced MRI increasing to 85% with the addition of contrast-enhanced sequences (Kataoka et al. [11]).

### Lymph node status and distant metastases

Recurrence in the inguinal lymph nodes carries a very poor prognosis, with most cases resulting in the patient’s death within 1 year; therefore, evaluation of nodal status at initial staging and adequate groin treatment is determinant for prognosis and overall survival [12].

First-line evaluation of inguinal lymph node involvement is clinical inspection and palpation, and a positive evaluation should prompt further imaging examination irrespectively of the primary tumour size and/or stromal invasion depth [5].

Several studies aimed to evaluate the accuracy of different imaging modalities (including MRI, ultrasound with or without fine-needle aspiration (FNA), CT and PET) in assessing metastatic involvement of inguinofemoral lymph nodes in vulvar cancer. (The results of these studies are summarised in Table 1.)

**Table 1:** The accuracy of imaging techniques in determining inguinofemoral lymph node metastases confirmed by histological examination

Imaging detection of inguinofemoral lymph nodes metastases							
Diagnostic investigation, study (year), [reference number]	Study design	Number of patients	Imaging modality	Imaging criteria	Sensitivity (%)	Specificity (%)	
Moskovic et al. [13]	Prospective	24	US	Round shape, or irregular configuration and loss of fatty hilum	85	83	
				US combined with FNA	83	82	
Hall et al. [14]	Prospective	44	US	Combination of lymph node size, shape, preservation/absence of an echogenic hilum, general attenuation and vascularity on Doppler	86	96	
				US combined with FNA	93	100	
Sohaib et al. [10]	Retrospective	21	MRI	Short axis ≥ 1 cm	40	97	
				Short axis ≥ 0.8 cm	50	100	
Hawnaur et al. [15]	Prospective	10	MRI	Long axis > 2, 1 cm, short axis > 1 cm, long-to-short axis diameter ratio < 1.3:1, irregular contour, and intranodal cystic changes	89	91	
Bipat et al. [16]	Retrospective	60	MRI	Combination of size (short axis), shape, contour, and aspect	52	85– 89	
Singh et al. [17]	Retrospective	39	MRI	Combined criteria (2 out of 3): short-axis > 1 cm; irregular or rounded shape; increased signal intensity on STIR or heterogeneous signal-intensity on T2-WI	85.7	82.1	
Kataoka et al. [11]	Retrospective	49	MRI	Short/long axis ratio ≥ 0.75	81.3	89.7	
				Contour	65.6	75.0	
				Necrosis	37.5	93.1	
				Loss of fatty hilum	75.0	72.4	
				Similarity of SI with primary tumour	82.1	60.0	
				Readers’s confidence of metastasis	87.5	86.2	
Cohn et al. [18]	Prospective	15	PET	FDG uptake	67	90	
Hullu et al. [19]	Prospective	25	PET	TYR uptake	75	62	
Crivellaro et al. [20]	Prospective	29	PET/CT	FDG uptake	53	85	
Andersen et al. [21]	Prospective	27	CT	Short axis > 1 cm or abnormal pattern of CE	60	90	
Pounds et al. [22]	Prospective	116	CT	–	59.1	77.8	
Bohlin et al. [23]	Retrospective	134	CT	Short axis > 1 cm or abnormal shape, attenuation or CE	Tumours < 4 cm (*n* = 87)		
					17	95	
					Tumours ≥ 4 cm (*n* = 47)		
					67	100	

US—Ultrasound, FNA—fine needle aspiration, STIR—short tau inversion recovery, SI—signal intensity

For all imaging modalities, the most commonly used criterion for regional lymph node metastasis is the short-axis, usually considered suspicious when > 1 cm; however, its reported sensitivity is low, ranging between 43 and 86% [11, 16, 17, 23, 24]. Other features may be helpful, especially when combined, namely irregular contour, round shape, presence of necrosis, loss of fatty hilum and a ratio of short-to-long-axis diameter ≥ 0.75. Lymph node necrosis demonstrated the highest specificity among individual criteria; however, it has low sensitivity [11, 12]. Care should be taken when MRI is performed shortly after a diagnostic vulvar biopsy, as this may result in reactive lymph node changes that may be mistaken by metastatic lymphadenopathy yielding a false-positive result [15]—awareness of this possibility and consultation of the cytological/histological results from the (recently) biopsied lymph node should be sufficient to avoid this misdiagnosis.

Several studies have analysed the added value of CT in the staging of primary vulvar cancer. In four prospective studies [21, 22, 24, 25] that aimed to investigate if preoperative CT influences surgical treatment planning, the authors concluded that preoperative CT scanning is of limited value and has no clinical impact as a routine examination, suggesting that it may be omitted in early stage vulvar cancer. On the other hand, in cases of locally advanced disease or in the presence of pathologically proven tumour spread to the inguinal or iliac lymph nodes, further staging with contrast-enhanced CT of chest, abdomen, and pelvis may provide valuable information and is recommended [5]. Within the major studies [16, 19], the coverage of the CT scans varied, including either the abdominal region or the chest and abdominal regions, but always including the pelvic and inguinal regions. All CT scans were performed with contrast enhancement.

### Sentinel lymph node biopsy

Traditionally, groin treatment in early stage vulvar cancer has included inguinofemoral lymph node dissection (IFLD), which involves the removal of superficial inguinal and deep femoral lymph nodes. While this is an effective approach in promoting survival, it carries a significantly higher risk of complications (such as lymphedema) with increased short- and long-term morbidity when compared to debulking of clinically involved lymph nodes or sentinel lymph node (SLN) [20]. Since only 25–35% of women with early stage vulvar cancer have groin metastases, IFLD may be considered an overtreatment in most of these cases [21].

In order to avoid unnecessary IFLD, several prospective multicentre trials have evaluated the safety and validity of SLN procedure in early stage vulvar cancer. A multicentre observational study [22] was conducted on 403 women who had primary vulvar tumours with less than 4 cm in size and depth invasion of more than 1 mm—inguinofemoral lymphadenectomy was performed only in patients with a positive SLN. With a median follow-up period of 35 months (24-month minimum), groin recurrences were detected in six of the 259 patients (2.3%) with negative SLN and the 3-year survival rate was 97%. There was a significant reduction in short- and long-term morbidity in cases where only the SLN was removed in comparison with SLN removal followed by IFLD. The long-term follow-up of the GROINSS-V observational study [23], which was also performed on this cohort, compared the results of SLN-positive patients (followed by IFLD) with SLN-negative patients (no IFLN dissection) in a total of 377 patients. At a median follow-up of 105 months, they found no significant differences (*p* = 0.03) in the overall local recurrence at 5 years (24.6% for SLN-negative and 33.2% for SLN-positive patients) and at 10 years (36.4% for SLN-negative and 46.4% for SLN-positive patients). Isolated groin recurrence rate was 2.5% for SLN-negative patients and 8.0% for SLN-positive patients at 5 years. Disease-specific 10-year survival was 91% for SLN-negative patients compared to 65% for SLN-positive patients (*p* < 0.0001).

A systematic review and meta-analysis [24] of the cumulative data on SLN biopsy in women with unifocal tumours measuring less than 4 cm and without clinically suspicious inguinofemoral nodes found no significant differences in the rate of groin recurrence after SLN biopsy (3.4%) in comparison with complete IFLD (1.4%). In addition, a recent systematic review by a European expert panel [25] concluded that SLN correlates with a low groin recurrence rate and a good 5-year disease-specific survival rate in negative SLN patients, and therefore SLN is currently considered the standard procedure in well-selected women with clinically unsuspicious lymph nodes.

## ESUR guidelines

For primary tumours ≤ 2 cm, confined to the vulva and/or perineum, and with ≤ 1 mm of stromal invasion, imaging staging is not recommended. Pelvic MRI including the inguinal regions should be performed for local staging of SCC with stromal invasion > 1 mm, tumour size > 4 cm, or tumours with suspicious involvement of the urethra, vagina, or anus according to clinical evaluation. For tumours > 2 cm and ≤ 4 cm, clinical staging and groin ultrasound (with puncture of suspicious lymph nodes) or MRI staging are both considered valid options.

For regional or locally advanced disease (FIGO stages III–IVA) or suspicious distant metastases (FIGO stage IVB), chest, abdominal and pelvic CT (or PET/CT) with coverage of the inguinal regions should be performed. Intravenous contrast should be administrated with image acquisition on portal-venous phase (60–80 s) to increase diagnostic accuracy.

The MRI recommendations on imaging of primary vulvar SCC are given in Table 2. Fasting and administration of anti-spasmodic agents are recommended, similarly to the ESUR guidelines for other gynaecologic conditions. The bladder should be emptied before imaging, since a fully distended bladder may inhibit both the degree of straining and the descent of pelvic organs [26]. Vaginal opacification with gel is optional. Future studies may help to establish the added value of vaginal gel in diagnosing small vulvar lesions and early vaginal invasion.

**Table 2:** Summary of the recommendations based on ≥ 80% agreement among experts

ESUR recommendations
**Recommendations for MRI staging of vulvar cancer**
**• Indications**
Tumour stromal invasion > 1 mm
Tumour size > 4 cm
Tumours with close proximity to or involvement of the urethra, vagina, or anus
**• Patient preparation:**
Fasting is recommended (4 – 6 h)
The use of antiperistaltic agents is recommended (20 mg butyl scopolamine IM/IV or 1 mg of glucagon IV) unless their use is contraindicated due to patient medical background
Supine patient positioning is recommended
Vaginal gel is optional
Rectal gel is not recommended
**• Hardware**:
The minimal recommended magnet field strength to stage vulvar cancer is 1.5 Tesla
**• Sequences and imaging planes:**
Pelvis
T1WI
Axial T1W Dixon sequence
T2WI
Axial, sagittal, and coronal two-dimensional T2W sequences
T2W sequence with fat suppression is optional
Slice thickness ≤ 4 mm
T2WI with a small FOV (from the vaginal top to the entire perineum included)
Axial or axial oblique (perpendicular to the urethra) and coronal or coronal oblique (parallel to the urethra)
Slice thickness = 3 mm is recommended
DWI-MRI
In the axial plane, with a minimum of two *b*-values (low *b* = 0–50 or 100 s/mm^2^, high *b* ≥ 800 s/mm^2^)
DCE-MRI
Three-dimensional (3D) spoiled gradient-echo fat-suppressed T1-weighted imaging (3D T1WI FS) imaging on axial or axial oblique before and after the administration of intravenous contrast for three scans to obtain arterial, portal and equilibrium phases (the last acquisition may be obtained in the most informative plane for each particular case)
Upper abdomen (to evaluate the Kidneys and lymph nodes)
T2W HASTE axial from the renal hila to the inguinal region
DWI axial from the renal hila to the inguinal region
**Recommendations for CT staging of vulvar cancer**
**• Indications**
Regional or locally advanced disease (FIGO stages III–IVA) or suspicious distant metastases (FIGO stage IVB)—alternatively to CT, PET/CT may be performed in these cases
**• Protocol**
Chest, abdominal and pelvic CT with coverage of the inguinal region after the administration of intravenous contrast with image acquisition on portal-venous phase (60 – 80 s)
**Recommendations for inguinofemoral lymph node US and biopsy**
**• Indications**
Ultrasound of the inguinal regions with biopsy of suspicious lymph nodes (either by FNA or core biopsy) should be performed in all patients with either clinical (palpation) or radiological suspicion of lymph node metastasis depicted on MRI, CT, or PET/CT

T2WI (T2-weighted imaging), DWI-MR (diffusion-weighted imaging magnetic resonance) and DCE-MR (dynamic contrast-enhanced magnetic resonance) are now recommended for the initial staging of vulvar cancers. Contrast-enhanced sequences depict vulvar cancers as early arterial enhancement lesions and can better delineate tumour invasion of the urethra, clitoris, vagina, or anus [12, 27]. Ideally, T2WI and DWI-MR should have the same acquisition plane, field of view, and slice thickness to allow side-by-side interpretation and/or image fusion as this improves diagnostic performance. T2WI with fat suppression may improve the detection of small tumours [2, 12, 27]; however, its usefulness is not consensual among experts, and therefore, it remains optional.

Further, T2WI sequences of the pelvis with a reduced field-of-view (rFOV) are advised since reducing the FOV increases spatial resolution and allows better anatomic detail, which may help in both the detection of small tumours and in delineating tumour invasion of nearby perineal structures [12]. These T2WI sequences with a rFOV may be obtained in axial oblique (perpendicular to the urethra) and coronal oblique (parallel to the urethra) planes.

For the benefit of spatial resolution with the possibility to reconstruct the acquired images in any desired plane, DCE sequences should be obtained using three-dimensional (3D) spoiled gradient-echo fat-suppressed T1-weighted imaging (3D T1WI FS) imaging on axial or axial oblique plane (perpendicular to the long axis of the urethra) on pre- and post-contrast administration, for three scans to obtain arterial, portal and equilibrium phases with the last acquisition obtained in the most informative plane for each particular case (usually in the sagittal or coronal plane to add a different perspective from the already acquired sequences with maximum resolution).

Imaging of the upper abdomen to evaluate the kidney and lymphadenopathy is recommended and should include T2W HASTE and DWI in the axial plane from the renal hila to the inguinal region.

### MRI structured report

Unanimous agreement was reached amongst panel members on the need for a structured MRI report in order to improve the report quality and to assist with the communication of clinically relevant information to the referring physician [28–32]. The recommended structured report is given in Table 3.

**Table 3:** Recommended MRI structured report in vulvar cancer staging

MRI reporting
Structured report is recommended and should addresses the following key points:
Tumour size (greatest dimension)
Tumour location (lateral, midline, multifocal)
Clitoris involvement when present
Relationship with adjacent perineal structures: urethra and vagina (lower third or upper part) and anus
Bladder/rectal invasion
Inguinofemoral lymph nodes status
Pelvic lymph nodes status
Other genital organs (uterus, cervix, vagina, and ovaries)

### Diagnosis and initial staging

Squamous cell carcinoma (SCC) is by far the most frequent malignant vulvar tumour. According to the latest World Health Organization (WHO) Classification of Tumours [33], SCCs must be classified on the basis of their association with the human papillomavirus (HPV) infection into SCC HPV-associated (having vulvar intraepithelial neoplasia (VIN) as a precursor lesion) or SCC HPV-independent (having differentiated VIN as a precursor lesion, often in association with lichen planus and lichen sclerosus). HPV-independent vulvar SCC has a worse prognosis than HPV-associated vulvar SCC, higher recurrence rates and a greater tendency to rapid progression [33]. In HPV-associated tumours, multifocal lesions and concomitant cervical neoplasia are more frequently observed [33, 34].

In most cases, patients are present at an early stage with vulvar tumefaction or ulcer that may be associated with pain, pruritus, bleeding, or discharge [27, 35]. Asymptomatic cases are less frequent. Diagnosis is histological and should be established with an incision biopsy [5].

MRI, ultrasound with or without puncture of inguinofemoral lymph nodes, CT and PET/CT may be used to define the extent of tumour and/or for treatment planning [11, 20, 21, 39, 40].

Lymph node biopsy may be performed either by FNA or by core biopsy. There are no published data comparing the performance of these to puncture techniques in the clinical setting of vulvar cancer staging, and the wider available experience addressing this topic comes from breast cancer studies [36–41]. In the study of Solon et al. [36], core biopsy of suspicious nodes showed a sensitivity rate of 96%, specificity of 100%, positive predictive value of 100%, and negative predictive value of 64%. All these data are superior to previously published studies on ultrasound-guided FNA, which have a sensitivity ranging from 50 to 80%. Moreover, false positive cytology and inadequate sampling are points of weakness of FNA. While the superiority of core biopsy over FNA in vulvar cancer staging has yet to be confirmed by specific prospective trials comparing these diagnostic techniques, in the author’s opinion, core biopsy should be preferred whenever possible to obtain sufficient material for histological analysis, although FNA can be considered appropriate for small suspicious lymph nodes.

The most widely used staging system for vulvar cancer is the one developed by the International Federation of Gynaecology and Obstetrics (FIGO) [42], which was revised in 2009 in close collaboration with the American Joint Commission on Cancer (AJCC) and the Union of International Cancer Control (UICC), and is given in Table 4. Major changes of this revision include the combination of the former stages I and II, subclassification of regional lymph node involvement based on the number and size of lymph nodes and the presence/absence of extra-capsular spread, as well as disregard for bilateral lymph node involvement [33]. These changes have been validated in several studies [3, 43, 44]. Complete staging using FIGO classification requires primary tumour resection and inguinofemoral lymphadenectomy; however, common practice has evolved to include the use of SLN biopsy as an alternative to complete lymph node dissection, as well as radiological assessment to determine local disease extension, with special emphasis to MRI [34, 45].

**Table 4:** FIGO 2009 classification for vulvar cancer staging

FIGO stage	Description
I	Tumour confined to the vulva (without nodal metastasis)
IA	Lesions ≤ 2 cm in size with stromal invasion* ≤ 1 mm
IB	Lesions > 2 cm in size with stromal invasion* > 1 mm
II	Tumour of any size with extension to the adjacent perineal structures (lower third of urethra, lower third of vagina, anus) without nodal metastasis
III	Tumour of any size, with or without extension to adjacent perineal structures (lower third of urethra, lower third of vagina, anus) with positive inguinofemoral nodes
IIIA	1. With 1 lymph node metastasis (≥ 5 mm), or
	2. With 1–2 lymph node metastases (< 5 mm)
IIIB	1. With 2 or more lymph node metastases (≥ 5 mm), or
	2. With 3 or more lymph node metastases (< 5 mm)
IIIC	With positive nodes with extracapsular spread
IV	Tumour invades any of the following:
IVA	1. Upper urethral and/or vaginal mucosa, bladder mucosa, rectal mucosa, or is fixed to pelvic bone
	2. Fixed or ulcerated inguinofemoral lymph nodes
IVB	Any distant metastasis including pelvic lymph nodes

^*^The depth of invasion is defined as the measurement of the tumour from the epithelial-stromal junction of the adjacent most superficial dermal papilla to the deepest point of invasion

Typically, initial treatment of vulvar cancer consists of complete surgical excision, with or without adjuvant radiation therapy (RT) and/or chemotherapy depending on pathology and disease extension [33] (see section “Vulvar cancer: management and treatment” later on this article for a detailed discussion on treatment planning and current guidelines).

### FIGO stage I

**Fig. 2:**
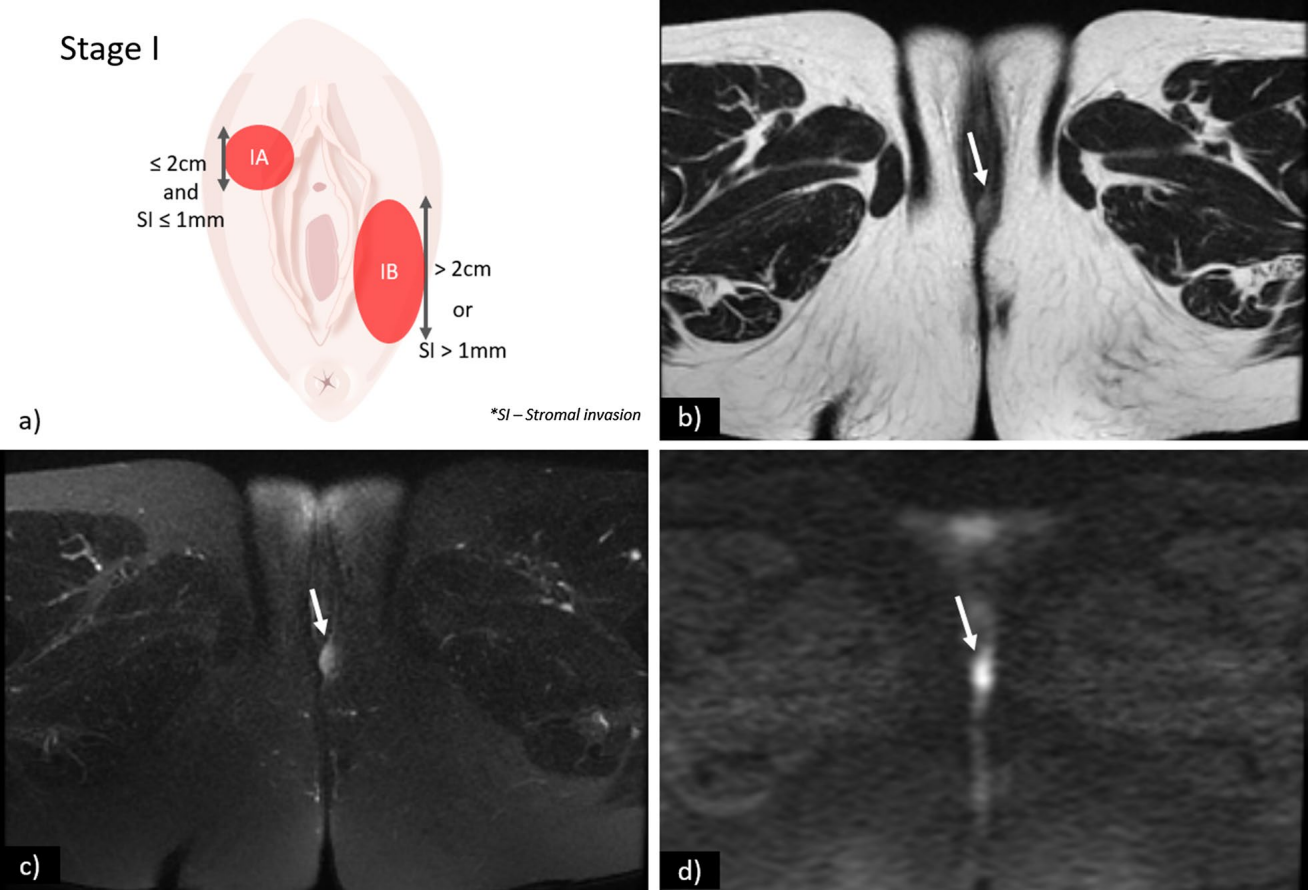
**a** Schematic illustration of FIGO stage I. Axial T2WI (**b**), axial fat saturation T2WI (**c**) and DWI with *b*-value = 800 s/mm^2^ (**d**) shows a vulvar tumour measuring < 2 cm, with pathologic proven stromal invasion of 4 mm, corresponding to FIGO stage IB. SI—Stromal invasion

Stage I is defined as a tumour confined to the vulva or perineum without lymph node or distant metastasis. It is further sub-divided into stages IA and IB according to tumour size and stromal invasion (Fig. 2):

- **Stage IA**—Lesions ≤ 2 cm in size with stromal invasion ≤ 1.0 mm.
- **Stage IB**—Lesions > 2 cm in size or with stromal invasion > 1.0 mm.

**Fig. 3:**
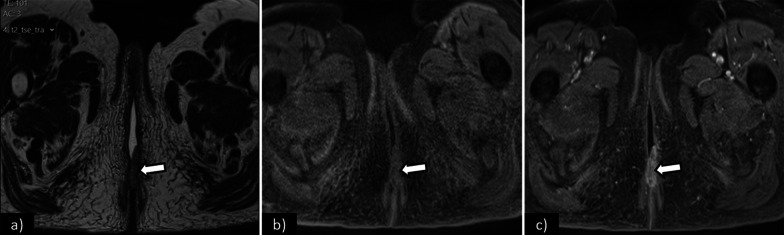
Axial T2WI (**a**), axial T1WI fat saturation before (**b**), and after gadolinium (**c**) shows a vulvar tumour measuring 2.5 cm, corresponding to FIGO stage IB. Note the increased conspicuity of the tumour in the contrast-enhanced sequence (**c**)

The role of imaging is limited in stages IA and IB. Vulvar carcinoma is depicted as a solid mass with nonspecific low signal intensity on T1WI and intermediate to high signal intensity on T2WI. DWI-MRI demonstrates restricted diffusion as a high signal intensity lesion on high *b*-value images with low signal intensity on the corresponding apparent diffusion coefficient (ADC) maps. DCE-MR imaging sequences with early arterial phase tumour enhancement may be useful in the detection of small vulvar lesions [12] (Fig. 3). T2WI with fat suppression may also be a helpful sequence, as the perineal region is rich in fat with high-signal intensity on T2WI, and its suppression may make small vulvar lesions more conspicuous [2, 12, 27].

### FIGO stage II

**Fig. 4:**
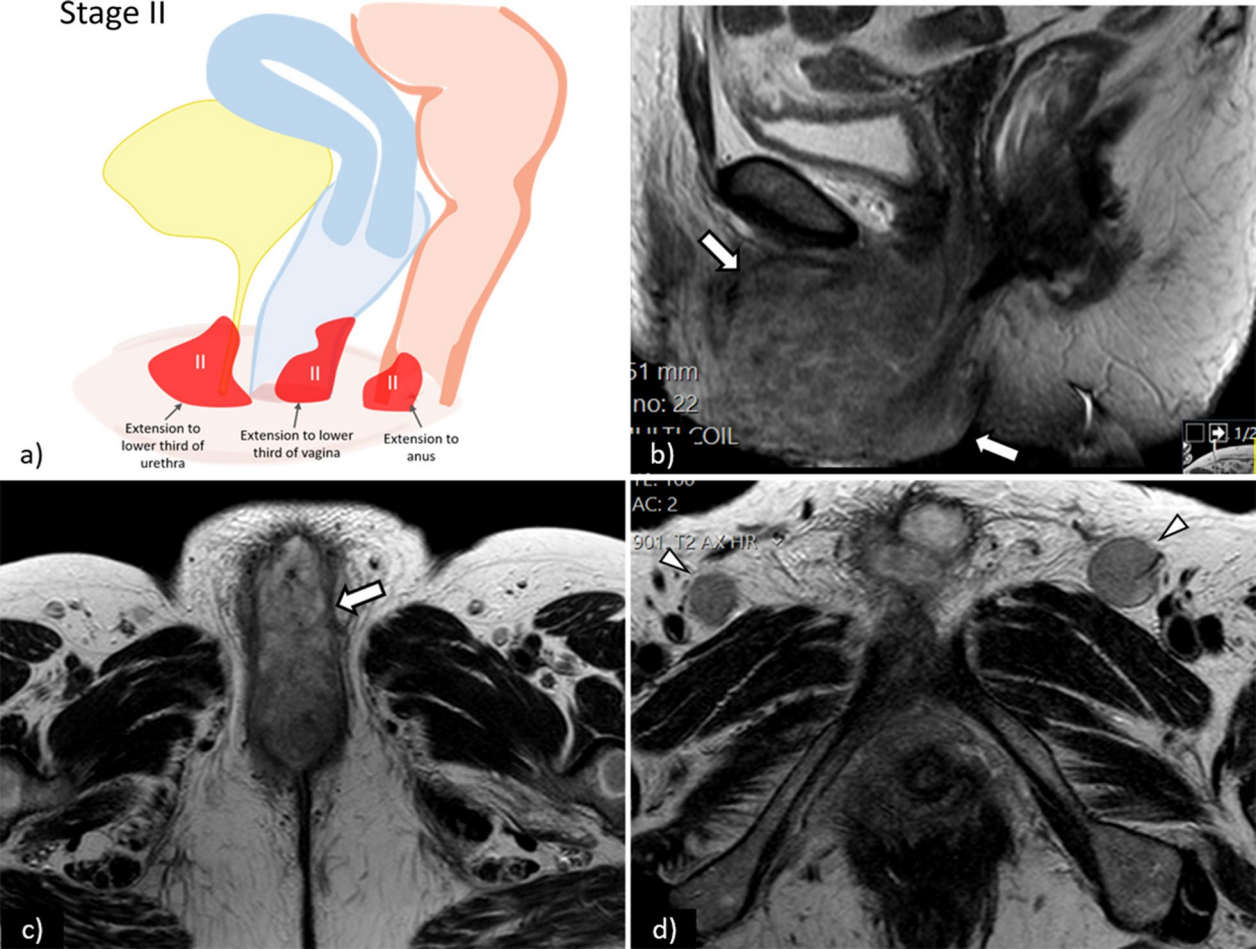
**a** Schematic illustration of FIGO stage II. Sagittal (**b**) and axial (**c**) T2WI of the pelvis show a large tumour (arrows) with invasion of the lower third of the urethra—FIGO II. Axial T2WI of the groins (**d**) shows bilateral enlarged inguinofemoral lymph (arrowheads) proved to be reactive on cytology

Stage II is defined as a tumour of any size with extension to adjacent perineal structures (1/3 lower urethra, 1/3 lower vagina, anus) without lymph node or distant metastasis (Fig. 4)**.**

**Fig. 5:**
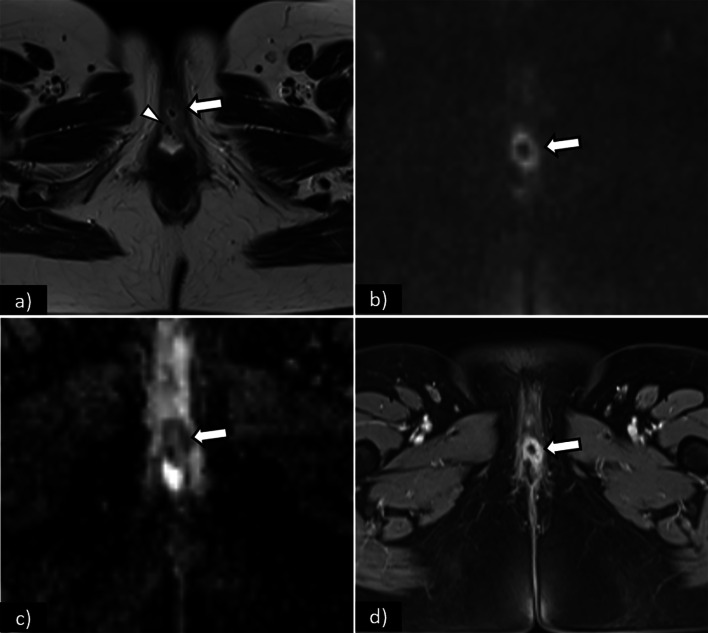
Axial T2WI (**a**) shows an intermediate signal intensity tumour (arrow), measuring 3 cm, with central necrosis and invasion of the external urethral meatus (arrowhead)—FIGO II. On DWI, the tumour is depicted by a high-signal intensity lesion on DWI (*b*-value = 1000 s/mm^2^) (arrow), and low-signal intensity on the corresponding ADC map (arrow). On T1WI fat saturation contrast-enhanced sequence (**d**), the tumour shows early arterial enhancement of its solid component (arrow), with no enhancement of the central necrotic portion. Note the increased conspicuity of the lesion in DWI-MRI (**b**) and **c** and in DCE-MRI (**d**)

On T2WI sequences, local tumour invasion may be depicted by disruption of the hypointense signal that circumscribes the urethra and/or interruption of the low signal intensity of the vaginal wall or the anal sphincter by an intermediate to high signal intensity tumour [27]. Large tumours may demonstrate high signal intensity on T2WI sequences due to internal necrotic changes [12]. DCE-MRI increases the staging accuracy and can better demonstrate involvement of the urethra, anus, and vagina [11, 27] (Fig. 5).

### FIGO stage III

**Fig. 6:**
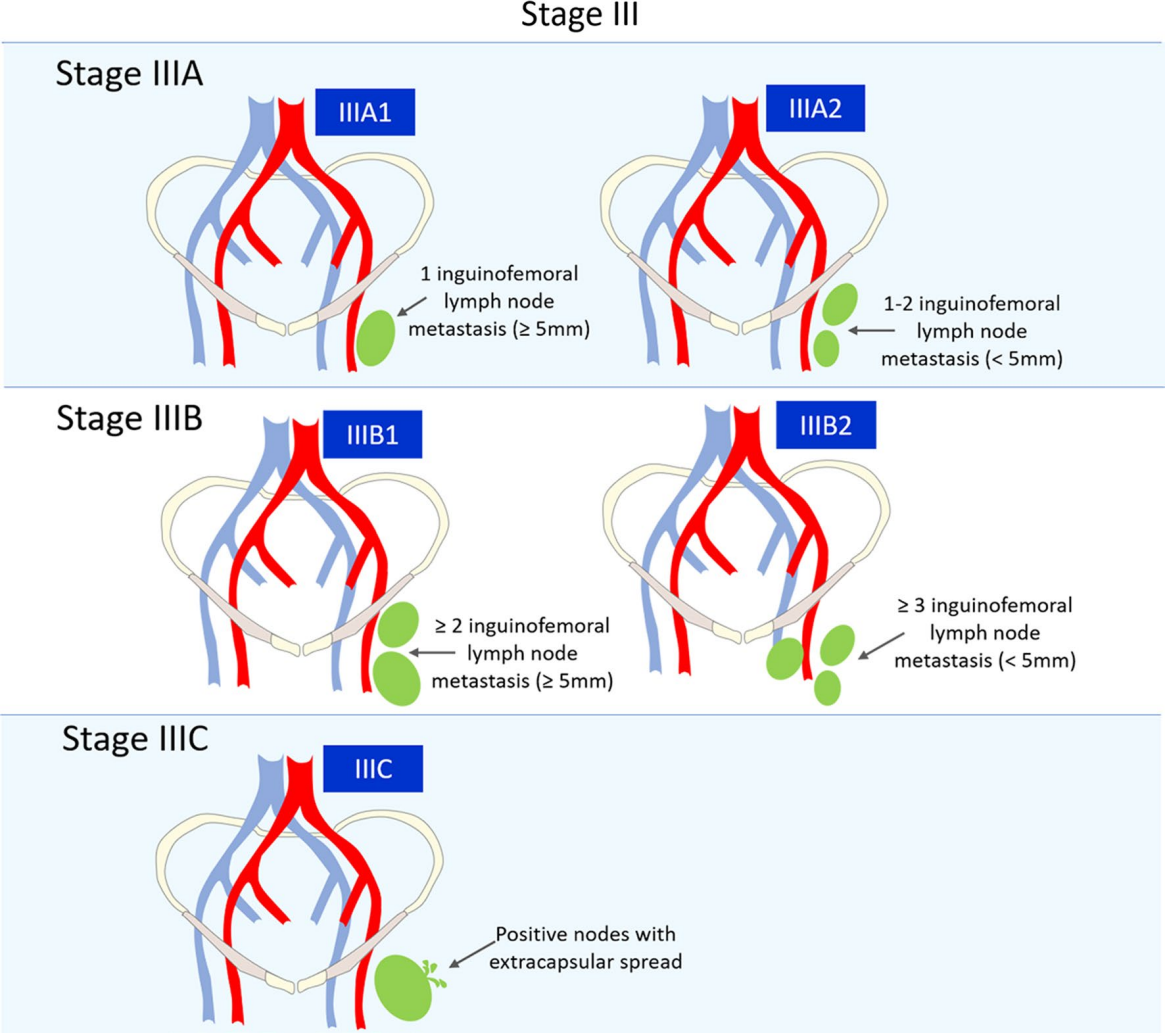
Schematic illustration of FIGO stage III

Stage III represents inguinofemoral nodes involvement irrespective of tumour size or local extension. It is further subdivided according to the number and size of the lymph nodes involved, as well as the presence/absence of extracapsular spread (Fig. 6). This last criterion is a result of the significantly worse prognosis of node metastases with extracapsular spread, which are associated with a five-year overall survival of 34% versus 66% in patients with intranodal metastases [44].

- **Stage IIIA1**—1 lymph node metastasis (≥ 5 mm).
- **Stage IIIA2**—1–2 lymph node metastasis(es) (< 5 mm).
- **Stage IIIB1**—2 or more lymph nodes metastases (≥ 5 mm).
- **Stage IIIB2**—3 or more lymph nodes metastases (< 5 mm).
- **Stage IIIC**—Positive nodes with extracapsular spread.

The risk of lymph node metastases is associated with primary tumour size, depth of stromal invasion, and the presence of lymphovascular space invasion. Vulvar carcinoma spreads via the lymphatic system primarily to the superficial inguinal nodes, as well as to the deep inguinal nodes (also known as deep femoral nodes), which are considered as regional sites. The subsequent involvement of pelvic lymph nodes is considered as distant metastasis. Lateral vulvar carcinomas drain to the ipsilateral inguinal lymph nodes, although lesions at or within 1 cm of the midline can drain to one or both sides [12, 27].

Pelvic lymph nodes are rarely involved in the absence of ipsilateral inguinal lymph node involvement, and an exception is made to some midline vulvar carcinomas and tumours with invasion of the vagina, bladder, or anus (above the dentate line) that may rarely spread directly to the pelvic lymph nodes (via the internal pudendal chain and internal iliac chain) [7].

Regional lymph node metastatic spread is the most important prognostic factor in vulvar cancer and determines the treatment choice [34, 46]. As part of the latest revision in the FIGO staging system, not only the number of metastatic lymph nodes, but also metastasis size and the presence/absence of extra-nodal spread, should be stated by the pathologist.

**Fig. 7:**
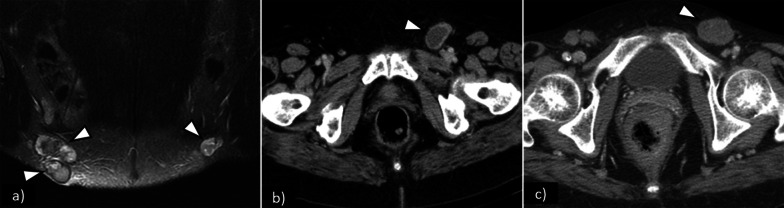
Pathologically proven inguinofemoral lymph node metastasis in different patients (FIGO III): **a** coronal fat saturation T2WI shows bilateral enlarged heterogeneous lymph nodes with necrotic changes depicted by intra-nodal high-signal intensity areas; **b** axial CT shows left inguinofemoral enlarged node with low-attenuation necrotic centre; **c** axial CT shows heterogeneous enlarged left inguinofemoral node

**Fig. 8:**
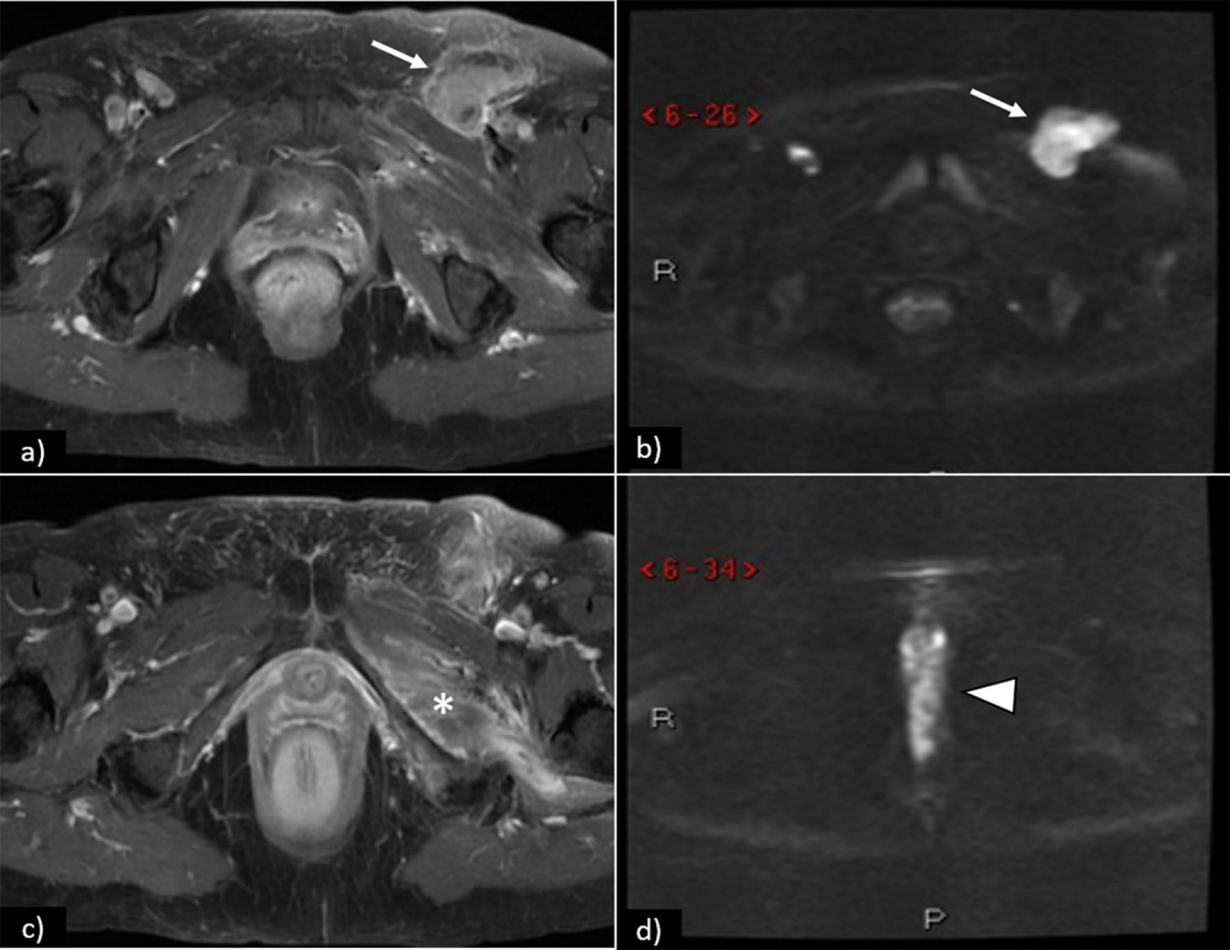
Axial contrast-enhanced T1WI **a** shows left inguinal lymphadenopathy (arrow) with restricted diffusion (arrow) on DWI (**b**). At different levels, axial contrast-enhanced T1WI (**c**) depicts left external obturator muscle involvement (*), and DWI (**d**) shows restricted diffusion of the primary vulvar tumour (arrowhead)

The most well-accepted MRI criterion for regional lymph node metastasis is short axis > 1 cm. Other features may be helpful, especially when combined, namely:irregular contour, round shape, presence of necrosis, loss of fatty hilum, and a ratio of short-to-long-axis diameter ≥ 0.75. Some of these features are shown in Figs. 7 and 8.

In case of discrepancy between positive radiological findings for inguinal lymph node metastasis (depicted at MRI, US, CT or PET/CT) and negative cytological/histological results following biopsy, the FIGO stage cannot be certainly estimated, and the multidisciplinary board must deliberate the most appropriate management for each case. If there is a strong radiological suspicion, a second ultrasound-guided lymph node biopsy may be performed using fusion virtual navigation systems that fuse real-time ultrasound images with previously acquired cross-sectional images using CT, MRI, SPECT/CT or PET/CT [47–49].

### FIGO stage IV

Stage IV comprises locally or regionally advanced disease (IVA) and distant disease (IVB).

**Fig. 9:**
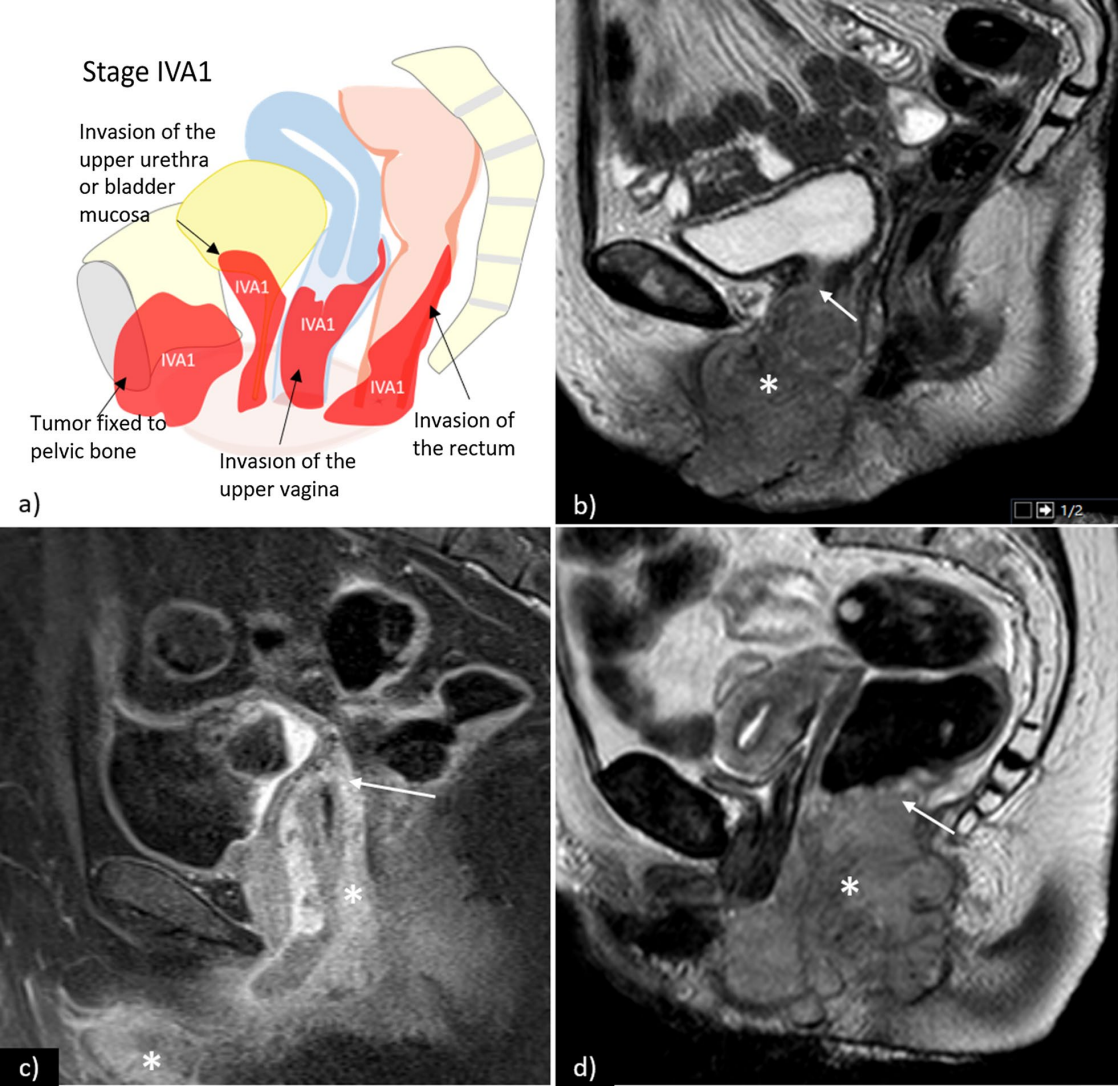
**a** Schematic illustration of FIGO stage IVA1. Examples of stage IVA1 vulvar carcinomas (*) in different patients: **b** sagittal T2WI sequences shows vulvar tumour with invasion of the upper third of the urethra (arrow); **c** sagittal contrast-enhanced T1WI sequence shows vulvar tumour with invasion of the upper third of the vagina (arrow); **d** sagittal T2WI sequences shows vulvar tumour with invasion of the rectum (arrow)

**Fig. 10:**
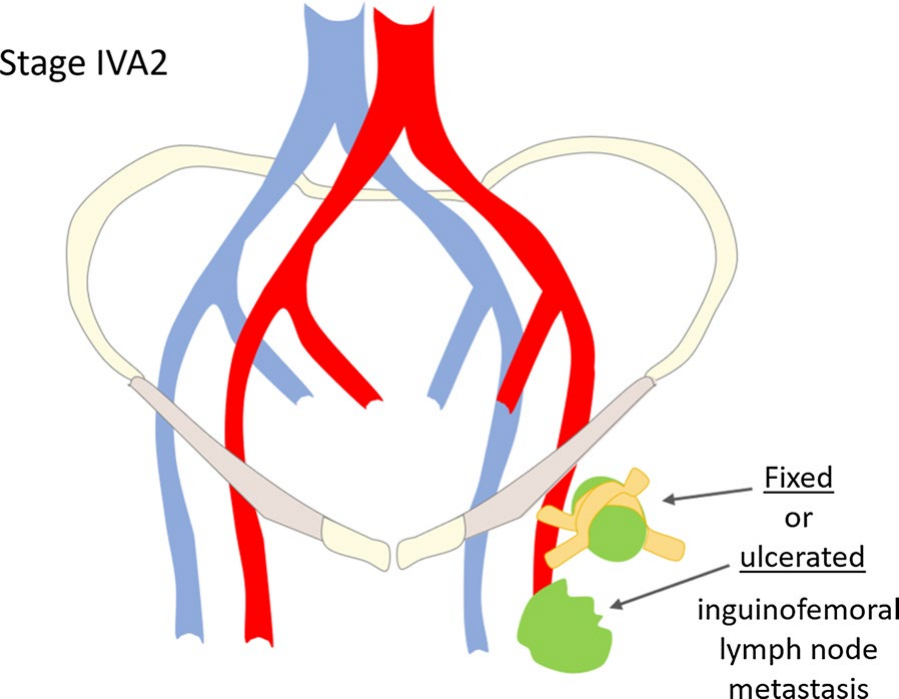
Schematic illustration of FIGO stage IVA2

**Fig. 11:**
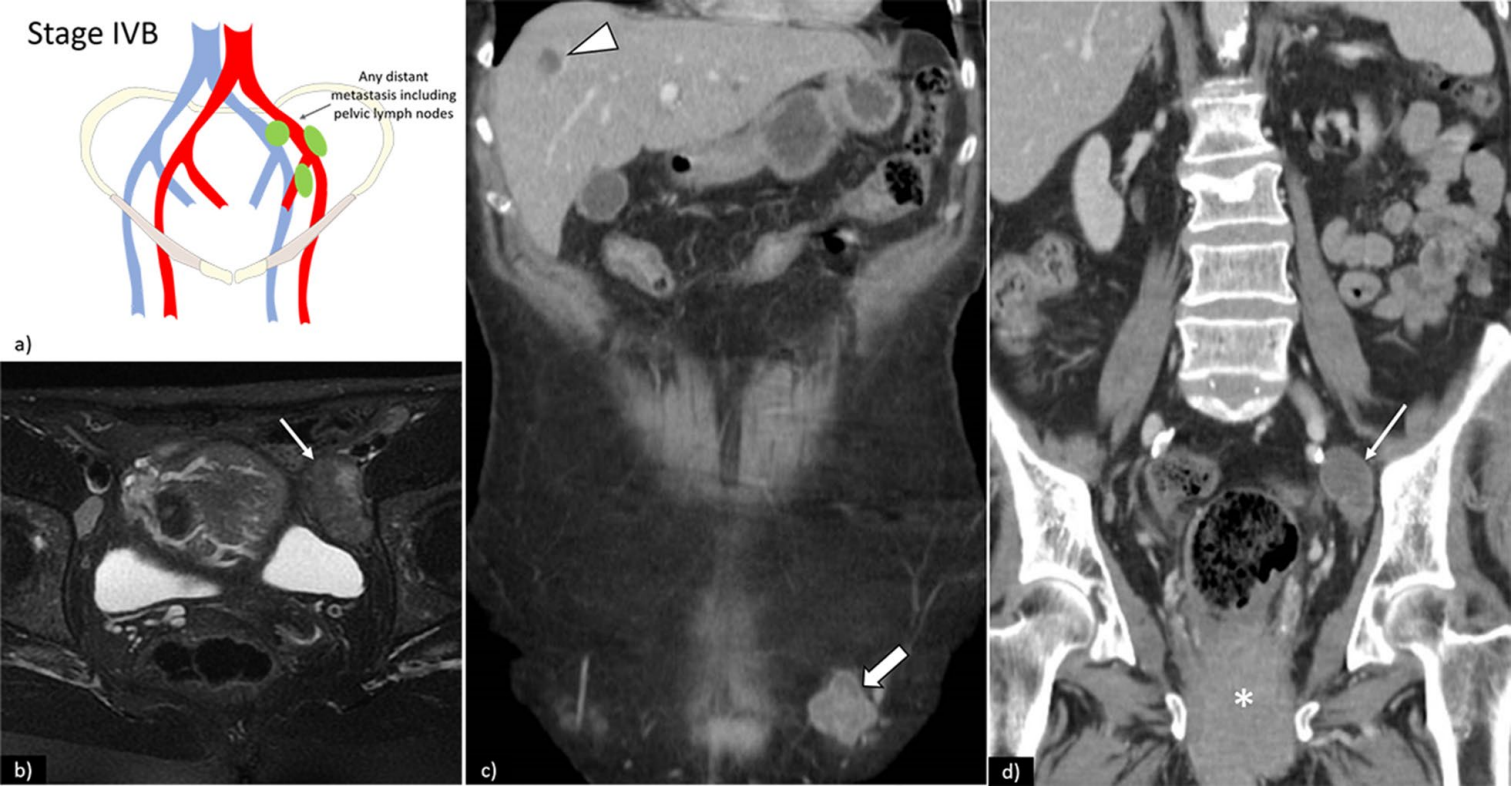
**a** Schematic illustration of FIGO stage IVB. Axial fat saturation T2WI shows left external iliac lymph node metastasis (arrow), depicted by increased lymph node size and heterogeneity. In another patient, coronal CT (**c**) and (**d**) shows vulvar tumour (*) with inguinal lymph node involvement (open arrow), internal iliac lymph node involvement (arrow), and hepatic metastasis (arrowhead)

- **Stage IVA1—**tumour invades upper 2/3 of urethra and/or vagina, bladder mucosa, rectal mucosa or is fixed to pelvic bone (Fig. 9).
- **Stage IVA2—**fixed or ulcerated inguinofemoral lymph nodes (Fig. 10).
- **Stage IVB—**any distant metastasis, including pelvic lymph nodes (Fig. 11).

Similar to FIGO stage II, invasion of regional pelvic structures can be depicted by an intermediate-signal intensity on T2WI disrupting the low-signal intensity of the upper two-thirds of the vagina or the upper two-thirds of the urethra. Invasion of the bladder/rectal mucosal is diagnosed if an intermediate signal intensity tumour on T2WI disrupts low-signal intensity bladder or rectal wall and extends into the mucosa or the lumen [27]. DCE-MR can assist in better delineating tumour invasion of the nearby structures [12].

Distant metastasis is a rare occurrence in vulvar cancer, often preceded by one or more local recurrences. Most frequently involved sites include lung, liver, bone, lymph nodes (axillary, thoracic, and paraaortic), and skin [46, 50]. In these cases, the prognosis is very poor with a two-year overall survival rate of 11.3% and a median survival from first diagnosis of metastases of only 5.6 months [50].

### Vulvar cancer: management and treatment

Clinical and radiologic assessment of the inguinal regions (either by ultrasound, CT, PET/CT, or MRI) are needed to detect possible metastatic lymph node, which should then be analysed by FNA or core biopsy whenever this additional information impacts the primary treatment choice. Locally or regionally advanced-stage disease (with histologically proven regional lymph node metastasis) should be further staged with contrast-enhanced CT of the thorax, abdomen, and pelvis [5].

#### Local treatment

Local treatment of early stage vulvar carcinoma consists of radical local excision [5].

Surgical excision margins of at least 1 cm are advised. In cases of close proximity between the tumour and the clitoris, urethra, or anus, smaller margins may be considered in an attempt to preserve their function. If surgical margins are close (< 8 mm) or positive, a second resection should be attempted. In cases of persistent positive margins or if the patient is not eligible for a second surgical intervention, adjuvant local RT is advised [5, 33, 51].

Treatment of advanced-stage vulvar cancer involves multiple treatment modalities including surgery, radiotherapy (RT), and chemotherapy. The optimal choice should be discussed in a multidisciplinary setting [5, 33].

#### Groin treatment

IFLD is not required for stage IA disease due to its low risk of lymph node metastasis [5, 52–55].

For tumours greater than stage IA (i.e. with stromal invasion > 1 mm) groin treatment should be performed. Depending on tumour size, SLN procedure (for tumours < 4 cm) or IFLD (for tumours ≥ 4 cm or in case of multifocal disease) is recommended. In cases of a positive SLN with a node metastasis < 2 mm, RT has shown to be a safe alternative to ILFD [56].

Contralateral IFLD may be performed when there is ipsilateral node involvement [5]. Postoperative RT to the groin is advocated for cases with more than 1 metastatic lymph node and/or in the presence of extracapsular lymph node spread [5].

#### Unresectable disease

In advanced-stage unresectable disease (larger stage II and stage IVA tumours), definitive chemoradiation is the treatment of choice. In selected cases, neoadjuvant chemoradiation should be considered [5].

#### Recurrent disease

Recurrences of vulvar carcinoma are common and usually occur within the first 2 years after initial presentation [12]. Vulvar and perineal region are the most frequent sites of local recurrences. Life-long follow-up after primary surgical treatment is advised and includes clinical examination of the vulva and groins (despite the low sensitivity of palpation in identifying groin metastasis, since available data does not support the routine use of imaging of the groins in follow-up) [5]. Clinical suspicion should be followed by biopsy and imaging work-up [57].

Local recurrences should be treated as primary tumours with wide local excision and inguinofemoral lymphadenectomy (if not previously performed), with or without postoperative radiotherapy [5]. CT of the chest and abdomen or PET/CT is recommended to assess the presence of additional metastases [5]. MRI is useful to examine the extent of the local recurrence and to plan further treatment.

In groin recurrence, restaging by CT (or PET/CT) of the chest, abdomen and pelvis is advocated and the preferred treatment is radical excision (when possible), followed by adjuvant radiation in radiotherapy-naive patients. When surgery is not possible, definitive chemoradiation is recommended [5].

If distant metastases are present, systemic (palliative) therapy should be considered along with local radiotherapy for control of locoregional disease [33].

## Summary

The authors’ recommendations on the initial staging of vulvar cancer are in accordance with the latest revision of the FIGO classification (2009). These ESUR guidelines were developed by the Female Pelvis Imaging Working Group, with the main purpose of standardising MRI protocols, interpretation, and reporting, ultimately aiming to reduce ambiguity and improve the contribution of radiology in the staging and management of these patients.

## Data Availability

Not applicable.

## Abbreviations

3D T1WI FS: Three-dimensional spoiled gradient-echo fat-suppressed T1-weighted imaging
ADC: Apparent diffusion coefficient
AJCC: American Joint Commission on Cancer
DCE: Dynamic contrast-enhanced
DWI: Diffusion-weighted imaging
ESGO: European Society of Gynaecological Oncology
ESUR: European Society of Urogenital Radiology
FDG-PET: 18F fluorodeoxyglucose positron emission tomography
FIGO: International Federation of Gynaecology and Obstetrics
FNA: Fine-needle aspiration
FOV: Field-of-view
HPV: Human papillomavirus
IFLD: Inguinofemoral lymph node dissection
rFOV: Reduced field-of-view
RT: Radiotherapy
SCC: Squamous cell carcinoma
SLN: Sentinel lymph node
SPECT/CT: Single photon emission computed tomography/computed tomography
T1WI: T1-weighted imaging
T2WI: T2-weighted imaging
TNM: Primary tumour (T), regional lymph nodes (N), distant metastases (M)
UICC: Union of International Cancer Control
VIN: Vulvar intraepithelial neoplasia
WHO: World Health Organization
